# Dietitians’ endeavor to contribute to the nutritional health of children and youth with intellectual disability and autism

**DOI:** 10.1080/20473869.2023.2283645

**Published:** 2023-12-20

**Authors:** Päivi Adolfsson, Jennie Hysing, Pia Ek

**Affiliations:** 1 Department of Public Health and Caring Sciences, Health Equity and Working Life, Uppsala University, Uppsala, Sweden; 2 City of Stockholm, Sweden; 3 Department of Medical Biochemistry and Microbiology, Uppsala University, Uppsala, Sweden

**Keywords:** Habilitation, transition, Sweden, nutritional advice, teamwork collaboration adolescent, coming of age, autism spectrum disorder

## Abstract

The aim of the present study was to explore the experiences of registered dietitians (RD) who consult children and youth with intellectual disability (ID) and autism. Another aim was to investigate how knowledge and working methods were transferred to RDs working with adults with ID and autism. Twenty-six RDs completed a web-based study-specific questionnaire with multiple-choice and open-ended questions. The respondents’ comments and responses to the open-ended questions were analyzed using systematic text condensation. The analyses resulted in four categories: Reachability and accessibility of RDs, *Clients do not comply with RDs’ dietary advice*, *RD finds individual solutions* and *Better collaboration for better knowledge*. It was noteworthy that RDs’ undergraduate education did not prepare them for clients with ID and autism. Instead, they learned by doing and from other professionals at the clinic if they collaborate with them or were part in teams around the client. The RDs reported a lack of national routines for the transition process of nutrition support from young to adult.

## Introduction

Individuals with intellectual disability (ID) and autism spectrum disorder are at a greater risk of developing health problems caused by their living conditions than people in general (Kolset *et al.* 2018, Zwack *et al.* 2022). Comorbidity in these individuals is high already from birth and increases more rapidly with age compared to other persons (Hirvikoski *et al.* 2021, Slevin *et al.* 2014, WHO 2010). Because of their disabilities, they depend throughout life on support from formal and informal caregivers (WHO 2010). These health problems also influence the wellbeing of the parents (Peña and Payne 2022). Children and youth with ID and autism need the same access to health care as other children but may also need access to specialist treatment and care (Kolset *et al.* 2018). Therefore, caregivers should initiate efforts early to reduce the risk for development of e.g. metabolic disorders such as diabetes, cardiovascular disease, overweight, obesity and underweight (Kolset *et al.* 2018, WHO 2010). These efforts include early nutritional support that can initiate and preserve a healthy lifestyle for the remainder of the individual’s life (Kolset *et al.* 2018). It is well-documented that overweight and obesity are more common in children and adolescents with ID (Ptomey *et al.* 2020, Slevin *et al.* 2014) than in children and adolescents in general (Maïano *et al.* 2016).

In Sweden, supporting health problems for this group of persons is provided mainly through habilitation centers. The children at risk for ID and autism are detected at birth, in preschool, at regular medical examinations or in school. The child is registered at a habilitation center when a diagnosis is set. The support in the habilitation center is organized differently for children compared to adults, mainly because it is more regular and frequent for children (Umb-Carlsson 2008). Habilitation teams are at best organized around them in a person-centered context. The aim is to give clients professional support to increase or maintain their abilities (Umb-Carlsson 2008). A registered dietitian (RD) is a professional certified to support clients with nutrition-related health problems (Wikström 2008). In habilitation centers, RDs’ role is to consult both children and adults to decrease the risk of nutritional comorbidity.

Persons with ID and autism need transition from health care units/supporting teams for children to adult healthcare units/supporting teams. It happens that they by themselves or their significant others (e.g. relatives, staff and trustees) are not aware of this change in support. Internationally, there are reports on the need for and how to prepare these children and their caretakers when they become adults (Fremion *et al.* 2022). The few studies concerning the transition from childhood to adulthood for persons with ID and autism show that the transition is a difficult period for both individual and family (Brown *et al.* 2019, Hergenroeder *et al.* 2015, Lahaije *et al.* 2023, Roos and Søndenaa 2020). Cooperation is needed in transition processes between the stakeholders using a person-centered approach (Benson *et al.* 2021). To facilitate the transition, checklists should be used in the healthcare system that includes all persons involved early in the transition process (Fourmaux *et al.* 2021).

Adolfsson *et al.* (2019) reported how RDs in Sweden experienced their professional role when working with adults with ID. Shortcomings described were that they were consulted when the problems were already ongoing for some time, supporting staffs’ insufficient knowledge in nutrition often resulted in low compliance for their advice and there were too few RDs in the organization. Roesler and Probst (2023) showed that weight issues, limited food choices, numerous persons involved in food, cooking and feeding of the client and lacking communication abilities were some barriers for dietary behavior change the RDs stated when they consulted these clients. Besides, the RDs express a need for adjustments in the education of RDs to improve their skills in working with clients with ID and autism (Adolfsson *et al.* 2019, Roesler and Probst 2023). The present study explored the experiences of RDs who consult children and youth with ID and autism. A further aim was to investigate how knowledge and working methods were transferred to RDs working with adults with ID and autism.

## Method

The current study used a web-based questionnaire including multiple choice and open-ended questions to examine the experiences of RDs working with the nutritional health care of children and youth with ID and autism. Systematic text condensation (Malterud 2012) was used to analyse the open-ended questions and comments in the questionnaire. The Swedish Ethical Review Authority approved the study (Dnr 2021/00367).

### Respondents

To enrol RDs with experience counselling children and youth with ID and autism all Swedish regional habilitation centers (*n* = 21) were contacted by e-mail to gather information about RDs employed within the centers. Fifteen centers responded positively, and 42 RDs were identified in these regions. Two habilitation centers replied that services to children and adolescents with such needs were offered at rehabilitation clinics instead. They passed our information to these clinics resulting in two more RDs. In parallel, 22 RDs with interest in consulting children and adults with ID and autism were identified in a network organized by the Swedish Association of Clinical Dietitians. A third approach was contacting children’s hospitals by e-mail; three RDs were found using this approach. In total 69 RDs were identified for inclusion.

### Questionnaire

A study-specific questionnaire about RDs’ working routines and experiences, used in an earlier study directed to RDs consulting adults with ID (Adolfsson *et al.* 2019), was used after some adjustments. The adjustments were to add parent and school as alternatives in some of the multiple-choice questions and add new questions about the transfer RDs experienced when consulting children *vs.* consulting adults. The first questions in the questionnaire were about the respondents’ sex, clinical affiliation, professional experience as an RD in general and the length of experience in working with children with ID and autism. The following questions focused on RDs’ clinical work consulting children with ID and autism: For what reasons do clients and their significant others contact an RD? Who initiates the first contact? What are the usual consulting procedures? What barriers and facilitators do the RDs experience in their effort to achieve satisfying results through consulting? Additionally, there were two questions about routines for clients’ transition from child to adult care. The respondents were asked to describe the routines and how they organized the transition when there were no routines. Including space for comments in multiple-choice questions and using open-ended questions were done to ensure respondents could provide information unforeseen by the researchers and inspired by the novelty of the research area (Patton 2015).

### Procedure

The questionnaire was administered using a survey tool. Together with information about the study, a link to the questionnaire was distributed to the 69 identified RDs by e-mail. The information included assurances that participation was voluntary, that the questionnaire was anonymous and that all information provided by respondents would be treated confidentially. Anonymity was guaranteed by the survey tool. Five reminders were sent out before the link to the questionnaire was closed after 5 wk.

### Data analysis

The respondents’ answers to the open-ended questions and comments were analyzed with systematic text condensation (Malterud 2012), a thematic cross-case analysis composed of four steps inspired by phenomenological ideas. The material included two meaning units, which were analyzed separately. The first included the respondents’ comments and replies about their general work consulting children and youth with ID and autism. The second meaning unit included the content of the two replies about the routines of the transition to adult care.

The analyses of the first meaning unit (Part one) started by combining all open-ended answers and comments belonging to the meaning unit into a document, which all authors then read to obtain an overview. Thereafter, the authors discussed the content of the material. Four preliminary themes emerged from the material about the work and working conditions of RDs, which the authors agreed upon. Next, the first author preliminarily organized the meaning units, coding the units and sorting the codes into code groups according to the preliminary themes. The first and second authors discussed the codes and code groups, and re-coding was performed until a consensus was reached. Each code group was considered an analytic unit, and the codes were sorted into subgroups. After that, the content of the meaning units was condensed. Finally, the phenomena of each theme with the most salient content and meaning were determined. All three authors participated in this step. The subgroups and code groups were then used to build new subcategories, and categories were labelled with headings illustrating their content.

The analysis of the second meaning unit (Part two), Transition procedure, which was shorter than the first, included responses about the transition from child to adult care. The responses were combined into a document and read by all authors. The authors had a mutual understanding that the sorting needed to be made according to how respondents described the transition. The first author performed the sorting operation. The results were then discussed and agreed upon by all authors.

## Results

Twenty-six (38%) of the 69 RDs invited to the study completed the questionnaire. They all worked in habilitation centers, so 62% of all identified at this working place answered the questionnaire. All respondents answered the open-ended questions and frequently commented. The results are illustrated with quotations, showing that they are expressed by respondents with different work experience with children with special needs in years in brackets. Table 1 shows the respondents’ working experience.

**Table 1.** Respondents’ working experience.

	MD	Range	Mean
Work experience as an RD	8.0	2.0–33.0	11.4
Work experience as an RD before consulting children with special needs	2.5	0–30.5	5.6
Work experience of consulting children with special needs	4.0	0.5–22.0	5.8

### Part one

**Figure 1.**
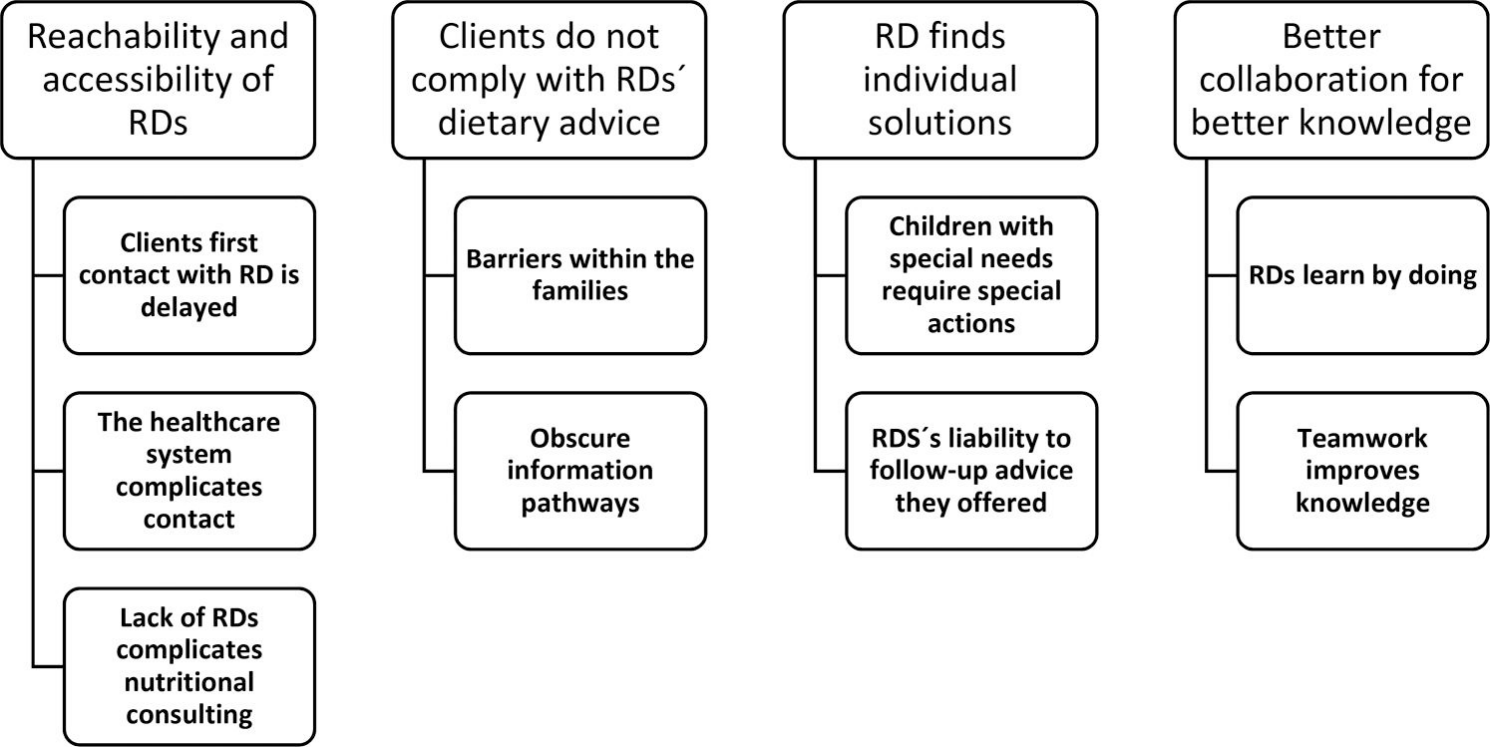
Categories and subcategories for part one of the analyses.

The categories that emerged from respondents’ answers and comments are shown in Figure 1.

#### Reachability and accessibility of RDs

The first category includes issues with the initial contact channels between clients and RDs. The category consists of the procedures within the habilitation centers that hinder contacts and inaccessible RDs within the habilitation centers.

### Clients first contact with RD is delayed

The respondents expressed that getting in contact with an RD can be difficult for clients. RDs commonly meet clients first when their nutritional problem has become severe or has been ongoing for some time. Only 4 of 26 (15%) respondents considered that they were involved in the clients’ nutritional problems at an early stage.

It varies, I sometimes wonder why it takes so long to involve an RD at obesity. (3 years’ experience)

The respondents stated that the main task of RDs at the habilitation centers was to consult clients with underweight. Other professionals consult clients with other nutritional problems at an early stage, and clients with overweight are often referred to a special overweight unit. The respondents noted that the most common diagnosis of their clients was underweight (23 of 26), followed by overweight and obesity (15 of 26) and food selectivity (14 of 26). They also reported consulting clients with gastrointestinal problems and conditions requiring nutrition supplements.

I only meet them when they are underweight, but others can hear from for instance a speech therapist or a nurse. (8 years’ experience)

### The healthcare system complicates contact

Respondents emphasized initial contact between the RD and client is first taken when the problem reaches a more severe level because a client or a relative cannot book an appointment with an RD at the habilitation center (i.e. a referral is required). The referral is written by other professionals at a habilitation center, often by a physician. The respondents indicated other professionals may not have good insight into nutrition and might not always appreciate the unique needs of a client with ID or autism.

My cases should always come from the team. The team does not always leave it to me, they believe that I am too busy to engage in “simple” questions like selective eating or prolonged meal time – that of course can develop to more serious problems. (22 years’ experience)

The respondents stated that parents tend to address the child’s food-related problems when in contact with habilitation center. However, the respondents pointed out that other professionals might sometimes neglect such parental concerns regarding food intake of a child with ID.

There is often an anxiety for the food intake among caregivers that is not taken seriously by people around including the health care professionals. (2 years’ experience)

### Lack of RDs complicates nutritional consulting

The respondents described a lack of RDs within the habilitation centers. Nutritional consulting by specialists is not prioritized at the centers, and centers are unwilling to increase the hours for RD service. Instead of increasing RD resources, habilitation centers feel that initial nutritional counseling should be with a nurse. Therefore, the workload of RDs is not designed to consult new clients and work preventively with dietary problems (such as being overweight). The respondents noted that not having time to meet clients and their families as soon and as often as needed could be stressful. They stated some families need extra support involving school staff, which can be stressful for an RD with no time for additional support. When a habilitation center does not offer the services of an RD for some time, the absence of an RD can lead to long waiting times and distress. When the service comes on stream again, it can be impossible for the RD to work preventively.

Unfortunately there is not enough time or resources to meet some patients as rapid and often as there is a need for. (3 years’ experience)

#### Clients do not comply with RDs’ dietary advice

The second category deals with an RD’s difficulty in conveying dietary advice to clients, their families and other persons significant for their nutritional health.

### Barriers within the families

The RDs encountered difficulties when providing families with dietary advice. According to the respondents, complications in consulting can be caused by parents’ cognitive disabilities or they have problems understanding the language. Such complications can make it challenging for the clients to interpret and understand the information mediated during counseling. The respondents further explained that implementing dietary advice can be problematic for families. Moreover, some parents have their approach to nutrition and what to eat. Thus, these parents do not want to follow the RDs’ advice.

Difficulties to understand the language, often misunderstandings in connection to interpretation to home language. This in combination with low intellectual level of the informal caregiver. Informal caregivers who have their own view on how the nutrition should be handled and do not want to follow advices or ordinations. (10 years’ experience)

The respondents also commented that complex family relationships, or other problems within the family, complicate a child’s nutrition plan. They explained the families could experience extreme stress to cope with everyday life, making it even more challenging to follow nutritional advice. Additionally, the respondents said they understand the difficulty of making behavioral changes related to nutrition and that theoretical plans do not always work in the daily life of families.

The difficulties around changes in behavioral. In principle, all human beings have difficulties for changes, including changes in dietary habits. I can have a splendid plan, theoretically, but it is more easily said than done to get adherence. (3 years’ experience)

### Obscure information pathways

The respondents indicated that many other persons besides the family are involved in the child’s everyday life, including staff in school, ordinary health care and various kinds of supported housing. They underscored all clients receive the same information on nutritional advice. However, it can be difficult for an RD to reach everyone involved in nutritional advice or diet planning. The respondents said this problem is compounded by high staff turnover.

Changes in living/school staff. This often results in lack of communication and new significant others appear all the time that perhaps not get right information. (3 years’ experience)

The respondents explained that when the client or the family does not want others to know about the child’s nutritional problems, the work of the RDs gets complicated.

Secrecy. The parents, or the youth being of age, will sometimes not that we inform people around them. Misunderstanding and lack of support can then lead to that results are not achieved. (3 years’ experience)

Unclear communication pathways can also influence the contacts between the ordinary healthcare system and a habilitation center. The respondents felt that this complicates improving the children’s nutritional health. They explained that the best care for clients sometimes requires more than one care provider, not just the habilitation center. Therefore, clients are referred to a more suitable caregiver with the appropriate medical device. However, those caregivers are not always aware of the limited medical resources of habilitation centers and refer them back to the habilitation center before proper care has been initiated.

Difficulties in cooperation with colleagues in care. Our patients (with intellectual disabilities or autism, or who have a condition resembling autism) often need several caregivers and are often discriminated in common health care since they belong to “the habilitation”. This results in that the work at the habilitation center suffers, since we don’t work with medical issues. (3 years’ experience)

The respondents indicated that there could be leadership difficulties within a habilitation center and that clear directives or goals within the habilitation center are lacking. They declared it could be confusing for families to have contact with different professionals advising on different areas of the client’s life, advice that is sometimes contradictory. When several professionals are involved in a client’s consultation, it can be difficult for an RD to provide dietary advice.

Many professions give several advices, difficult to test everything in time, difficult to test new things on some patients. (3 years’ experience)

#### RD finds individual solutions

The third category deals with tactics RDs apply to consult children with special needs. Children and youth with ID and autism have unique and individual needs, and the RD has to support each client to make successful nutritional changes.

### Children with special needs require special actions

According to the respondents, the client and caregiver are commonly consulted at the habilitation center. When parents have difficulties bringing a child to the center, the consultation is conducted without the client. At other times, older children visit the RD alone when they do not want or need adult support.

The advice are relatively often given only to the caregivers or other responsible adult … It of course also depends on the age of the children and his/her level of cognition if the advices are given directly to the patient or to his/her relatives. (6 years’ experience)

The respondents considered home visits a priority practice to gain a better understanding of the client and family situation. During home visits, the RD can study the client’s daily meal situation, what type of food a client chooses and how the food is prepared. Gathering them all in the client’s home can be a good opportunity to spread the nutritional advice if a client has many care contacts.

When you need to see the patients in home environment to find out how the meal situation looks like. (0.5 years’ experience)

Despite the benefits of home visits, they are rarely a standard service. Twelve of 26 (46%) respondents never made home visits, and the rest reported that they occasionally made home visits. None of the respondents prioritized home visits because they were time-consuming.

It rarely happens due to lack of time since the dietitian resource is extremely small in comparison to the large coverage-area. (10 years’ experience)

In addition to home visits, telephone counseling can be a suitable and not-so-time-consuming alternative, especially when children are very small.

As the respondents pointed out, it is essential to communicate the nutritional plan to everyone involved in the child’s life (e.g. school, preschool or short-term supported housing) so everyone involved has the same information. However, they need approval from the parents or child to do so. Direct contact is taken when approved, or the RD can arrange meetings with them. It is also customary that parents themselves communicate the information about their child’s nutrition plan to everyone involved. The respondents described that RDs usually record, sometimes using easy-to-read texts with imageries, what has been decided during the consultation. This advice is then given personally to the family. Generally, the RD needs to remind the family about the nutrition plan.

### RD’s liability to follow-up advice they offered

Children with special needs require extensive support, and it is vital to evaluate the effects of the nutritional plan and, if needed, reevaluate earlier counsel. The respondents stressed that follow-up meetings are suitable (and necessary) until the client’s condition improves. Twenty-one of 26 (81%) respondents answered that they always conducted follow-ups, while the remaining answered that this was occasionally done. The respondents, who only met underweight clients, emphasized that it is crucial to follow the client’s growth curve and ensure that the client gains weight as planned.

Very important for evaluation of possible changed nutritional measures (10 years’ experience)

Respondents’ view about the responsibility of the follow-ups was two-fold. Some hold that the RD are responsible for follow-up the given advice and treatment to ensure the client’s safety. According to these respondents, RDs alone communicate the nutrition plan to everyone involved in the child’s life and how the plan is followed.

I believe that I as a dietitian has a responsibility to contact all involved and therefore it is also my responsibility to follow up. (3 years’ experience)

Other respondents believed that the *responsibility* of the nutrition plan lies with the families, who must decide when and if they want a follow-up. They also emphasized that it is time-consuming for an RD to contact everyone involved in the child’s life, prioritizing that, they will never have time to meet the clients for consulting.

It would of course had been best to get everything collected, partly to ascertain that everything get through but also to see if it is possible in every day work to do this. Nevertheless, it feels impossible to accomplish this with so many patients in one RD. I wouldn’t have time to give advice (3 years’ experience)

#### Better collaboration for better knowledge

The fourth category concerns RDs’ doubts about their education not preparing them for clients with special needs. An RD must actively search for adequate information and collaborate with other professionals to achieve the required knowledge for competent consulting.

### RDs learn by doing

The respondents believed they had a basic knowledge of the client group but wanted comprehensive education and training. They also felt they needed to learn more about the clients. The respondents said they gained much experience and expertise through their work. Only 1 of 26 (4%) respondents considered they received an adequate understanding of individuals with ID and autism through education.

Through work with persons in the client group. (17 years’ experience)

The respondents described collaboration that occurred at seminars and conferences arranged by habilitation centers or other organizations working with the client group. Regrettably, the respondents felt that few RDs focus on individuals with special needs.

Participating in network meetings for RDs who consult individuals with special needs is valuable for acquiring new experiences and knowledge. Some respondents reported having auscultated RDs at other clinics to get more experience.

To read about diagnosis, treatment, attend courses, attend conferences, seminars, network meetings, take part of others experience, talk to physicians, join teams and get the whole picture around the client. (12 years’ experience)

Moreover, the respondents emphasized they improved their knowledge about different diagnoses and treatments through professional journal articles and books.

### Teamwork improves knowledge

The respondents noted that gathering knowledge and experience in children with special needs and working with other professionals (nurses, physicians, speech therapists, occupational therapists and physiotherapists) at the habilitation center is helpful. They requested more cooperation with other professionals consulting the same clients. When working in a team, they felt they better understood a client’s condition, particularly concerning behavioral change.

You must take into account that it is a big task to make changes. It will be realized through experience, listening to the families, other co-working RDs (or physicians, speech therapists, nurses, psychologist). (8 years’ experience)

The RDs responded that, in addition to collaboration and teamwork at the habilitation center, to obtain further experience and knowledge, a mentor at the center could increase the RDs’ knowledge.

Experience and to consult experienced colleagues, primarily. (2 years’ experience)

### Part two

#### Transition procedure

The respondents observed the lack of national transition routines for clients who turn 18 and have to leave the habilitation center for children. Twenty of 26 (77%) respondents reported that their habilitation center had routines for the clients’ transition. Still, the routines vary depending on whether clients are transited within the habilitation center when they become adults or are transited to primary care units or specialized clinics.

Respondents working in habilitation centers serving children and adults reported that the transition was mostly smooth and the collaboration was well incorporated in the unit. RDs in child and youth units often work closely with the adult unit and share many common issues.

RDs for children and adults use the same clinic and it is easy to meet physically on a daily basis (7 years’ experience)

Transition routines in habilitation centers where the same RDs consulted children and adults are unnecessary as their clients could have the same RD in adulthood. However, the respondents remarked that some habilitation centers for adults consult only clients that use supplements for nutritional health. Clients with other conditions are transited to primary care clinics instead.

At enteral feeding: dietitians for children wright a final note and inform the dietitian for adults that the patient will be transferred either via internal message or phone. The others are transferred to primary care and for those a referral to primary care is written, where it is included that dietitian in primary care has to be put in, the dietitian for children writes the final note. (4 years’ experience)

The respondents explained that it is incredibly complicated when habilitation centers do not offer RD consultation to adults and transfer them to primary care. The respondents expressed concern about clients receiving the consultation they need after the transition, since they experienced problems finding adapted RD services within primary care.

A procedure exists concerning if a patient is referred to habilitation for adults, where there is an RD. There are no routines when a patient is referred to primary care where there is no accessible RD. (3 years’ experience)

Accordingly, the respondents mentioned that the transition to primary care could be considerably stressful for the whole family.

Use to call around to all possible dietitians in both inpatient care and primary care for help with the transfer. There is today no RD for adults that is responsible for the transfer of patients from the habilitation (CATASTROPHE and an extreme stress on the families. (2 years’ experience)

## Discussion

Our findings are similar to those in the study of adult support services offered by RDs (Adolfsson *et al.* 2019). Only RDs for children from habilitation centers participated, while RDs from habilitation centers and primary care clinics answered questions on adults with ID (Adolfsson *et al.* 2019). These findings, supported by a previous study (Umb-Carlsson 2008), indicate that in Sweden it is more common for children with ID and autism to be referred to habilitation centers than adults with ID and autism. A difference in responses from RDs working with children with ID and autism *vs.* those working with adults with ID is that the former expressed more concern over a lack of cooperation within the habilitation center. In contrast, RDs working with adults with ID were more concerned about the lack of collaboration with informal or formal caregivers outside of regular health care. Respondents in both studies wanted more client-oriented teamwork, consistent with the findings of Benson *et al.* (2021). Both studies describe problems at the organizational and resource levels.

The current results show that RDs working in habilitation centers meet the clients first when their nutritional problems are severe. Care teams, including habilitation centers, often comprise different health professionals (e.g. nurses, physicians, speech therapists, occupational therapists, dietitians and physiotherapists). In these teams, physicians and nurses are primarily responsible for the clients’ health. This hierarchy (Wikström 2008) is recommended by WHO (2010), suggesting that physicians and nurses should act as gatekeepers and guide clients to other professionals when necessary. Physicians and nurses offer dietary advice if they consider their nutrition knowledge is sufficient instead of referring the clients to a nutritional specialist (Pojednic *et al.* 2023, Wikström 2008). This may explain why RDs expressed that they were involved too late and why overweight and obesity are increasing in children and youth with disabilities, especially children with ID who need special care (Maïano *et al.* 2016, Ptomey *et al.* 2020, Slevin *et al.* 2014). However, the lack of RDs also compels the habilitation centers to send these clients to other clinics, where the reception is not adapted to treat these clients. RDs in the present study were frustrated because of limited resources, which prevented best practices from being followed. These issues were also described by Elliott and Gibson (2023).

The respondents represent RDs whose clients customarily have parents who meet their clients’ nutritional needs and who are often the recipients of nutritional advice. Thus, RDs’ nutritional advice are more or less modified and influenced by parents’ competence, attitudes, believes and complex family situations. Studies have also found that to create a stable relationship and attain successful results RDs should consider clients and their parents as experts and give individualized advice that can be implemented in the clients’ everyday life (Elliott and Gibson 2023, Holmberg Fagerlund *et al.* 2017, Peña and Payne 2022, Lahaije *et al.* 2023). RDs must also be sensitive to the family’s social and economic situation (Peña and Payne 2022, Lahaije *et al.* 2023). In addition, nutritional advice for child and adolescent clients must also be communicated to health professionals or providers that treat the same client. The respondents in our study had experiences with complications leading to contradictory advice, which can cause frustration for all those concerned. Appelgren *et al.* (2018) suggest a person-centred approach, that includes relationship between professionals and significant others, is needed to achieve successful and safe consultation.

In line with Lahaije *et al.* (2023) and Appelgren *et al.* (2018) our results show professionals should work in a person/family-centered manner to advise and highlight the need to be sympathetic to the everyday needs of family life. The problems of implementing nutritional advice to involved parties are discussed in Adolfsson *et al.* (2022). Home visits seem to be a useful strategy to exchange information, as seen in this and the Adolfsson *et al.* (2019) study, but are rarely done due to a lack of resources.

Although the RDs in our study had the same position, they had two distinct views associated with their role and liability to follow up on given advice: the principle to take responsibility for follow-ups or assign responsibility to the family. According to the professional certification (Wikström 2008), follow-up is the responsibility of the RDs. However, as we discussed earlier, RDs’ work is often affected by limited resources, which restricts performing the consultation according to expectations. Lack of time is a general problem for RDs in care delivery (Elliott and Gibson 2023). However, with follow-ups, an RD can develop a long-term relationship with a client based on trustworthiness and respect. Telephone follow-ups are usually less appreciated by clients and thus should not be a substitute strategy (Nagy *et al.* 2022, Elliott and Gibson 2023).

Clients with ID and autism are vulnerable and depend on health care professionals with good knowledge about their living conditions. The present study and the study of Adolfsson *et al.* (2019) suggest that RDs in Sweden need better knowledge about individuals with ID and autism. The significant others for these clients also expect RDs to have special skills they can apply (Adolfsson *et al.* 2022). The absence of knowledge about these clients is a major problem within different healthcare units (Appelgren *et al*. 2018, Roesler and Probst 2023).

Our respondents, all working at habilitation centers, described that working in teams with other professionals helps them gain knowledge and improves their overall skills. However, they did not always feel included in the team at their center. Wilson *et al.* (2019) reported that nurses feel they have authoritative knowledge about clients with complex needs, which the RDs may interpret to mean that their own knowledge is unnecessary. That RDs’ unique competence is not acknowledged might increase their feeling of exclusion (Wikström 2008). Nurses declare that collaboration with RDs is required but is difficult to accomplish because there are too few RDs to cooperate with at health care centers (Appelgren *et al*. 2022). Moreover, according to other health care professionals, collaboration with RDs is needed to offer clients suitable treatment in nutrition (Elliott and Gibson 2023) and is more often provided when RDs are included as equal partners in care teams (Pojednic *et al.* 2023).

Children and youth require repeated assessment of their needs and planned support to make successful and smooth transitions at each life stage (WHO 2010). There are typically more problems for persons with chronic disorders to become of age than for others (Fourmaux *et al.* 2021). In Sweden, persons with ID and autism should receive appropriate support in accordance with law (SFS 1993:387 2023), however as adults (≥18 years), they have to request support themselves. It might be that they do not apply for support because they do not know or are not aware that they still need support. The transition to adult nutritional consulting is a part of the transfer process that young persons must undergo. Hergenroeder *et al.* (2015), Benson *et al.* (2021), Fremion *et al.* (2022) and Ghanouni and Seaker (2022) report related problems and lack of appropriate transfer routines. Hergenroeder *et al.* (2015) note that transition guidelines should be present and used for planning before clients turn 18. The routines were also asked concerning the transition from children’s dietary counseling to counseling adults in the present study. Our results show a lack of routines for RDs during transition procedure for persons with ID and autism from childhood to adulthood. Some respondents felt the transition was a disaster. Many children with ID and autism lack dietary support when they are 18 or are told to contact primary care clinics, where functional knowledge is generally low (Appelgren *et al*. 2022). As indicated by our findings, there are no national routines for transition and not all habilitation centers have working routines for it. Lack of routines for the transition process is also a problem internationally where efforts are made to improve the transition. For that reason, Razon *et al.* (2019) tested a specific multidisciplinary transition team consult service and found that it significantly enhanced pediatric input-output capacity. Fourmaux *et al.* (2021) published a checklist that should be used to transfer children with chronic disorders to adult medical care in the USA. The Social Welfare Board in Sweden has clinical practice guidelines and regulations for different issues, but none for transfer routines for children with ID and autism becoming adults. Moreover, outside the healthcare system, there is not enough information on what happens at this age in the education given to children in the specialized elementary school or how adult living arrangements are organized, which worries their caregivers (Benson *et al.* 2021, Roos and Søndenaa 2020).

Sweden’s RD resources at habilitation centers for children and youth are limited. Nevertheless, 42 RDs working in habilitation centers were identified from the 21 regions of Sweden; of these, 26 (62%) participated in the current study, which should be a suitable representation. Because no national or regional register exists, there still is a risk that not all RDs were identified, still the RDs that were identified and participated in the present study have solid experience of both working as RD in general and with children with special needs (Table 1). However, in Sweden, children and youth with diagnose ID and autism are commonly referred to habilitation centers (Umb-Carlsson 2008). Adolfsson *et al. (*2019) showed that the RDs consulting adults valued the possibility of adding comments and detailed responses. Compared to interviews, a questionnaire with open-ended questions ensures that respondents are less affected by the interviewer and can articulate their experiences in their own words (Reja *et al.* 2003). All our respondents answered the open-ended questions and commented on the multiple-choice questions. Some responses were concise and direct but not less valuable than more elaborated responses, as seen from the respondent citations. However, the high response rate suggests that the RDs considered the study important. Thus, the information provided was considered sufficient for the aim of the study analysis. Furthermore, the questionnaire’s administration guaranteed the respondents’ anonymity *via* a survey tool. Systematic text condensation was used to analyse the answers to ensure the authors could approach the data with an open mind (Malterud, 2012). Because the study was performed in RDs working in Sweden with services for persons with ID and autism, the results may not be generalized to other populations or settings.

## Conclusions

Our findings and those of studies with adults present a similar picture of RDs working with persons with ID and autism. The results show that the perception that other professions can replace and do not ask for RDs’ professional competence, lead to a lack of RDs in Swedish health organizations. These are reasons why clients get in contact with an RD only after serious nutritional issues have emerged and hinder RDs from following up their nutritional advice. Lastly, knowledge of ID and autism is important, such knowledge is lacking in the Swedish educational system for RDs who learn by doing, especially if they are included in teamwork and collaboration with other professionals.

No national routines for the transition process of nutrition support exist when individuals with ID and autism turn 18. The respondents felt that local routines either do not exist or are not always well functioning especially not when the client is transferred from habilitation centers to primary care.

## Data Availability

The data that support the findings of this study are available from the corresponding author upon reasonable request.
